# (NH_4_)_3_B_11_PO_19_F_3_: a deep-UV nonlinear optical crystal with unique [B_5_PO_10_F]_∞_ layers

**DOI:** 10.1093/nsr/nwac110

**Published:** 2022-06-10

**Authors:** Bingliang Cheng, Zijian Li, Yu Chu, Abudukadi Tudi, Miriding Mutailipu, Fangfang Zhang, Zhihua Yang, Shilie Pan

**Affiliations:** CAS Key Laboratory of Functional Materials and Devices for Special Environments, Xinjiang Technical Institute of Physics and Chemistry of Chinese Academy of Sciences, Xinjiang Key Laboratory of Electronic Information Materials and Devices, Urumqi 830011, China; Center of Materials Science and Optoelectronics Engineering, University of Chinese Academy of Sciences, Beijing 100049, China; CAS Key Laboratory of Functional Materials and Devices for Special Environments, Xinjiang Technical Institute of Physics and Chemistry of Chinese Academy of Sciences, Xinjiang Key Laboratory of Electronic Information Materials and Devices, Urumqi 830011, China; Center of Materials Science and Optoelectronics Engineering, University of Chinese Academy of Sciences, Beijing 100049, China; CAS Key Laboratory of Functional Materials and Devices for Special Environments, Xinjiang Technical Institute of Physics and Chemistry of Chinese Academy of Sciences, Xinjiang Key Laboratory of Electronic Information Materials and Devices, Urumqi 830011, China; Center of Materials Science and Optoelectronics Engineering, University of Chinese Academy of Sciences, Beijing 100049, China; CAS Key Laboratory of Functional Materials and Devices for Special Environments, Xinjiang Technical Institute of Physics and Chemistry of Chinese Academy of Sciences, Xinjiang Key Laboratory of Electronic Information Materials and Devices, Urumqi 830011, China; Center of Materials Science and Optoelectronics Engineering, University of Chinese Academy of Sciences, Beijing 100049, China; CAS Key Laboratory of Functional Materials and Devices for Special Environments, Xinjiang Technical Institute of Physics and Chemistry of Chinese Academy of Sciences, Xinjiang Key Laboratory of Electronic Information Materials and Devices, Urumqi 830011, China; Center of Materials Science and Optoelectronics Engineering, University of Chinese Academy of Sciences, Beijing 100049, China; CAS Key Laboratory of Functional Materials and Devices for Special Environments, Xinjiang Technical Institute of Physics and Chemistry of Chinese Academy of Sciences, Xinjiang Key Laboratory of Electronic Information Materials and Devices, Urumqi 830011, China; Center of Materials Science and Optoelectronics Engineering, University of Chinese Academy of Sciences, Beijing 100049, China; CAS Key Laboratory of Functional Materials and Devices for Special Environments, Xinjiang Technical Institute of Physics and Chemistry of Chinese Academy of Sciences, Xinjiang Key Laboratory of Electronic Information Materials and Devices, Urumqi 830011, China; Center of Materials Science and Optoelectronics Engineering, University of Chinese Academy of Sciences, Beijing 100049, China; CAS Key Laboratory of Functional Materials and Devices for Special Environments, Xinjiang Technical Institute of Physics and Chemistry of Chinese Academy of Sciences, Xinjiang Key Laboratory of Electronic Information Materials and Devices, Urumqi 830011, China; Center of Materials Science and Optoelectronics Engineering, University of Chinese Academy of Sciences, Beijing 100049, China

**Keywords:** deep-ultraviolet, nonlinear optical materials, KBBF-like structure, fluoroborophosphate

## Abstract

Deep-ultraviolet (DUV) nonlinear optical (NLO) crystals that can extend the output range of coherent light below 200 nm are pivotal materials for solid-state lasers. To date, KBe_2_BO_3_F_2_ (KBBF) is the only usable crystal that can generate DUV coherent light by direct second harmonic generation (SHG), but the layered growth habit and toxic ingredients limit its application. Herein, we report a new fluoroborophosphate, (NH_4_)_3_B_11_PO_19_F_3_ (ABPF), containing four different functional units: [BO_3_], [BO_4_], [BO_3_F] and [PO_4_]. ABPF exhibits a KBBF-like structure while eliminating the limitations of KBBF crystal. The unique [B_5_PO_10_F]_∞_ layers enhance ABPF’s performance; for example, it has a large SHG response (1.2 × KDP) and a sufficient birefringence (0.088 at 1064 nm) that enables the shortest phase-matching wavelength to reach the DUV region. Meanwhile, the introduction of strong B-O-P covalent bonds decreases the layered growth habit. These findings will enrich the structural chemistry of fluoroborophosphate and contribute to the discovery of more excellent DUV NLO crystals.

## Teaser

This paper reports a new nonlinear optical crystal that has a large second harmonic generation response, a short cutoff edge and a sufficient birefringence for deep-ultraviolet phase matching.

## INTRODUCTION

Deep-ultraviolet (DUV) nonlinear optical (NLO) materials can expand the frequency range of all-solid-state lasers through cascaded second harmonic generation (SHG), which has important applications in lithography, semiconductor manufacturing and many other fields [1,2]. There are at least three basic requirements for DUV NLO materials: a large NLO coefficient (*d*_ij_ > 0.39 pm V^−1^) to improve laser conversion efficiency; a short cutoff edge in the DUV region (λ_cutoff_ ≤ 200 nm); and suitable birefringence (Δ*n*: 0.05–0.10) to meet the phase-matching (PM) condition in the DUV region [3,4]. These mutually constraining indicators (*d*_ij_, λ_cutoff_ and Δ*n*) are mainly determined by the electronic structures and microscopic properties (hyperpolarizability, highest occupied molecular orbital-lowest unoccupied molecular orbital (HOMO-LUMO) gap and polarizability anisotropy) of the anionic groups and their arrangements [5–7]. Unfortunately, a single anionic group can barely balance all three conditions due to the intrinsic limitations. In general, non-*π*-conjugated units, such as [BO_4_], [PO_4_] and [SO_4_], possess a large HOMO-LUMO gap that is beneficial to DUV transparency, however, their small polarizability anisotropy leads to small birefringence that cannot achieve the desired DUV PM property [8–10]. *π*-conjugated groups, [BO_3_], [CO_3_], [NO_3_], etc., have anticipated large optical anisotropy but the terminal oxygen atoms with dangling bonds harm the DUV transparency [11–15]. Based on these, several functional units with different utilities could be combined through some design strategies, such as chemical-substitution-oriented design, to obtain new structures that can fulfill expectations [16–19]. The well-known NLO material KBe_2_BO_3_F_2_ (KBBF), for example, can generate a DUV coherent light through direct SHG due to its unique two-dimensional (2D) [Be_2_BO_3_F_2_]_∞_ layers composed of [BO_3_] and [BeO_3_F] units. However, its layered growth habit, resulting from large interlayer spacing (6.25 Å), and the high toxicity of raw materials are not desirable [11].

Traditionally, the exploration of UV NLO crystals is mainly focused on borate and phosphate systems, and typical crystals include LiB_3_O_5_ (LBO), *β*-BaB_2_O_4_ (BBO) and KH_2_PO_4_ (KDP) [20–23]. Borophosphate, as a mixed-anionic system, is also a source of NLO crystals, and BPO_4_ (BPO) and MBPO_5_ (M = Sr, Ba) have been reported as NLO crystals with excellent properties [8,24]. Recently, we proposed a ‘fluorination strategy’ by substituting fluorine for oxygen atoms in borates to regulate the structure of NLO crystals, so as to achieve the balance of the three parameters mentioned above (i.e. *d*_ij_, λ_cutoff_ and Δ*n*) [5,6,25]. Also, this strategy was further extended to the phosphate system. Consequently, [BO*_x_*F_4-_*_x_*] (*x* = 1, 2 and 3) and [PO*_x_*F_4-_*_x_*] (*x* = 2, 3) units with superior microscopic properties were employed in NLO materials design, which led to the discovery of the promising NLO crystals: AB_4_O_6_F (A = NH_4_, Na, Rb, Cs), MB_5_O_7_F_3_ (M = Mg, Ca, Sr, Pb), (NH_4_)_2_PO_3_F, NaNH_4_PO_3_F·H_2_O, etc. [26–34]. However, fluoroborophosphate, as a system with even more functional anionic groups, has been left behind. To date, only 14 cases of fluoroborophosphates (organic–inorganic hybrids and mineral compounds are not included in the statistical data here) have been reported and deposited in the international inorganic crystal structure database (ICSD) [35–43]. As shown in Supplementary Table 1, non-*π*-conjugated units, including [BO_4_], [BO_3_F], [BO_2_F_2_], [PO_4_] and [PO_2_F_2_], construct the backbone of fluoroborophosphates. These compounds have large band gaps in the range of 4.34–6.45 eV, indicating their feasibility for applications in the UV or DUV region. Among them, five compounds are acentric and exhibit a moderate SHG response of 0.3–1.1 times that of benchmark KDP. Their birefringence (≤0.044) is not large enough to satisfy the DUV PM condition.

In this work, we attempt to introduce *π*-conjugated [BO_3_] units into the fluoroborophosphate system to enhance the birefringence, thus regulating the PM wavelength for DUV applications. A new fluoroborophosphate, (NH_4_)_3_B_11_PO_19_F_3_ (ABPF), with four kinds of structural units—[BO_3_], [BO_3_F], [PO_4_] and [BO_4_]—was successfully designed and synthesized. Fascinatingly, ABPF exhibits a new type of KBBF-like structure with unique [B_5_PO_10_F]_∞_ layers connected by shared oxygen atoms forming the final 3D framework. It inherits the excellent properties of KBBF, such as a wide transparency range, a large SHG response and a suitable birefringence to satisfy the DUV PM condition. Beyond these, ABPF has a non-layered growth habit and is chemically benign. These properties make ABPF a promising DUV NLO crystal. In addition, the contributions of multiple anionic groups to the linear and NLO properties of ABPF were confirmed by the first-principles calculations. Our results highlight the synergistic effect of multiple anionic groups on the design of DUV NLO materials and open up new possibilities for exploring DUV NLO materials in fluoroborophosphates.

## RESULTS AND DISCUSSION

Polycrystalline samples of ABPF were synthesized via the high-temperature solution method in a closed system, and the photograph of ABPF crystals is shown in Supplementary Fig. 1. Crystallographic data are contained in CCDC 2153289 in crystallographic information file format. The purity of the phase was checked by powder X-ray diffraction (XRD; see Supplementary Fig. 2). The results of thermogravimetric analysis-differential scanning calorimetry (TG-DSC) curves and powder XRD patterns show that ABPF begins to decompose after 180°C, and BPO_4_ was found in decomposition products (see Supplementary Figs 2 and 3). The constituent elements and the anion units are further confirmed by elemental analysis and infrared (IR) spectroscopy (see Supplementary Figs 4 and 5).

**Figure 1.**
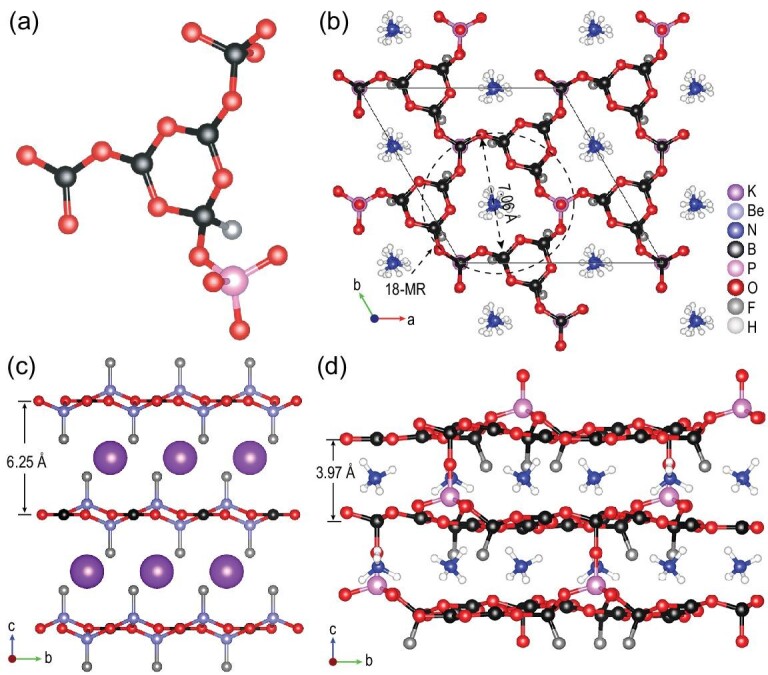
Crystal structures. (a) [B_5_PO_14_F] fundamental building block. (b) Two-dimensional [B_5_PO_10_F]_∞_ layers with 18-membered rings in the *ab*-plane. (c) Layer structure of KBe_2_BO_3_F_2_. (d) Crystal structure of (NH_4_)_3_B_11_PO_19_F_3_. Spacing of adjacent layers that pass through [BO_3_] units is 3.97 Å.

ABPF crystallizes in the trigonal space group *R*3 (see Supplementary Table 2), and the basic structure is shown in Fig. 1. Five crystallographically independent boron atoms exhibit three types of coordination environments, i.e. [BO_3_] triangle, [BO_4_] and [BO_3_F] tetrahedra, while one crystallographically independent phosphorus atom exhibits the coordination environment of [PO_4_] tetrahedron. The bond lengths, bond angles and bond valences are all in the reasonable range (see Supplementary Tables 3–8). The fundamental building block (FBB) is unique [B_5_PO_14_F], which is composed of the [B_3_O_6_F] ring and three branches: [PO_4_] tetrahedron, [BO_4_] tetrahedron and [BO_3_] triangle (Fig. 1a). Three FBBs are closed to form a large 18-membered ring (MR), and further polymerized to unprecedented 2D [B_5_PO_10_F]_∞_ layers extending in the *ab*-plane (Fig. 1b). Amazingly, similar layers with 18-MRs were also found in NH_4_B_4_O_6_F (ABF), and play important roles in its excellent NLO properties [26] (see Supplementary Fig. 6). Different from the 2D [B_4_O_6_F]_∞_ layers in the structure of ABF, the layers in ABPF are further connected by shared oxygen atoms of [BO_4_] and [PO_4_] tetrahedra, stacking along the *c*-direction to form a 3D framework. Also, the interlayer spacing of ABPF is 3.97 Å, less than that of KBBF (6.25 Å), and NH_4_^+^ cations are filled in the interlayer (Fig. 1c and d). Viewed along the *a*-axis, the [Be_2_BO_3_F_2_]_∞_ layers of KBBF are substituted by the [B_5_PO_10_F]_∞_ layers for non-toxicity and K^+^ cations are substituted by NH_4_^+^ cations for structural regulation, which results in the 3D framework of ABPF showing a KBBF-like structure. To the best of our knowledge, this is the first 3D KBBF-like structure with [PO_4_] and [BO_4_] tetrahedra bridging between layers. Compared with KBBF, the introduction of the strong B-O-P covalent bonds results in the reduction of interlayer spacing, which makes the interlayer interaction of ABPF significantly higher than that of KBBF, thus decreasing the layered growth habit.

**Figure 2.**
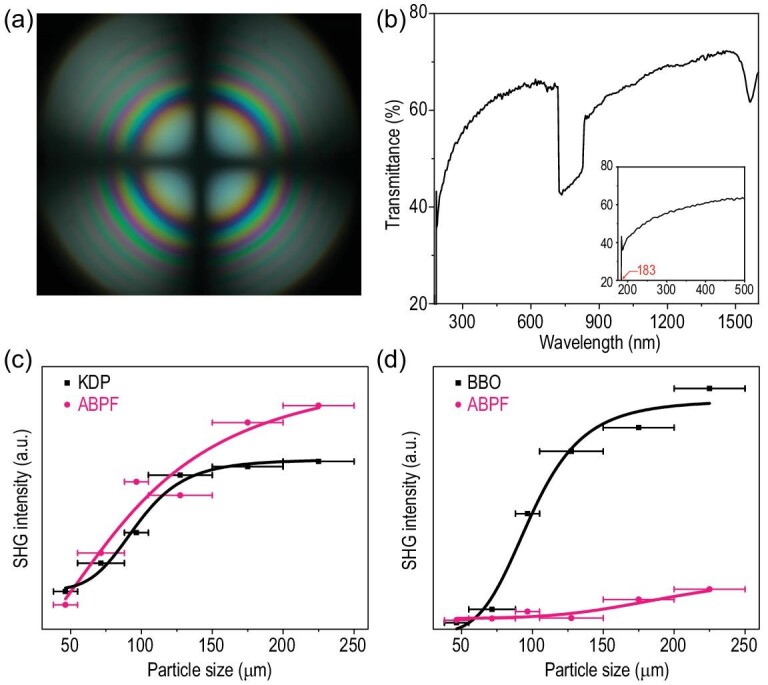
Experimental results. (a) Interference pattern of polarized light. (b) Transmittance spectra. Powder second harmonic generation (SHG) measurements at (c) 1064 nm and (d) 532 nm with benchmark KH_2_PO_4_ (KDP) and *β*-BaB_2_O_4_ (BBO) used as references.

The interference pattern of polarized light indicates that ABPF is a uniaxial crystal (Fig. 2a). The transmittance spectrum demonstrates that its UV cutoff edge is 183 nm (the corresponding band gap is 6.78 eV), indicating that ABPF has a wide DUV transparency window (Fig. 2b). Based on the charge-transfer model and Mulliken analysis [44,45], the bond valence of O atoms is in the range of 1.7–2.0 e (see Supplementary Fig. 7), which confirms that the introduction of non-*π*-conjugated [BO_4_] and [BO_3_F] units are beneficial to the partial elimination of the dangling bond, thus obtaining a DUV transparency. So, the short UV cutoff edge of ABPF is mainly attributed to the large HOMO-LUMO gaps of its microscopic anionic groups [5,6], [BO_4_], [BO_3_F] and [PO_4_] units, the elimination of the dangling bonds of [BO_3_] units, and the avoidance of unwanted *d*-*d* or *f*-*f* electron transitions by the selection of an A-site cation. Moreover, the SHG capabilities of ABPF were measured by the Kurtz-Perry method [46] under incident laser 1064 and 532 nm, respectively. Two standard NLO crystals, KDP and BBO, were used as the references. The output SHG response of ABPF is 1.2 × KDP at 1064 nm and 0.2 × BBO at 532 nm in the 200–250 μm particle size range, respectively (Fig. 2c and d).

**Figure 3.**
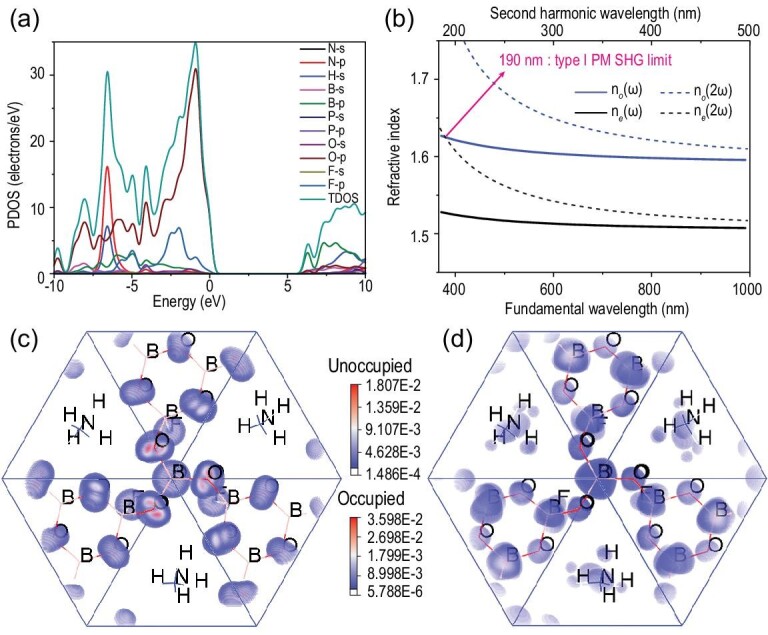
Calculation results. (a) Partial density of states (PDOS) of (NH_4_)_3_B_11_PO_19_F_3_. (b) Calculated type I phase-matching (PM) SHG limit. SHG density maps of the (c) occupied and (d) unoccupied orbitals in the virtual electron process of *d*_11_.

To further explore the structure–property relationship of ABPF, electronic structures and optical proprieties were calculated by the first-principles calculations based on density functional theory (DFT). The direct band gap of ABPF under a generalized gradient approximation (GGA) framework is 5.96 eV (see Supplementary Fig. 8), which is slightly smaller than the experimental value of 6.78 eV due to the discontinuity of exchange-correlation energy functional. To keep the band gap consistent with the realistic condition, a scissors operation (0.82 eV) was utilized when performing the optical properties calculations. From the partial densities of states (PDOS), the top of valence bands (VBs) and the bottom of conduction bands (CBs) are essentially dominated by O-2*p* and B-2*p* states, respectively (Fig. 3a). According to the Kleinman approximation of point group 3, there are four non-zero NLO coefficients for ABPF. The calculated values are *d*_11_ = 1.19 pm V^−1^, *d*_22_ = −0.91 pm V^−1^, *d*_31_ = 0.08 pm V^−1^ and *d*_33_ = 0.06 pm V^−1^, of which *d*_11_, *d*_22_ and *d*_31_ are in the effective NLO coefficient (*d*_eff_) expressions [47,48]. The largest tensor, *d*_11_, was analyzed by the SHG-density method to understand the contribution of NLO-active electron states and units. It shows that the virtual electron (VE) process is dominant in the SHG process, and the contributions of occupied states are mainly determined by the non-bonding O-2*p* and F-2*p*, while unoccupied states are mainly determined by the orbitals of B-2*p*, N-2*p*, O-2*p* and F-2*p* (Fig. 3c and d). In fact, the orbitals of non-centrosymmetric sublattices near the top of valence bands from [BO_3_] are mainly responsible for the SHG effect [49]. Meanwhile, the contribution origins of the SHG response were analyzed by the real-space atom-cutting method [48], and the result indicates that [BO_3_] units contribute most to the SHG response, while other units contribute relatively little (see Supplementary Table 9).

Suitable birefringence (Δ*n*) and mild dispersion are essential for realizing the PM conditions that foster a practical DUV laser output. The birefringence and PM wavelength were calculated by first-principles calculations based on DFT. To the best of our knowledge, ABPF has the largest birefringence of 0.088 at 1064 nm among all fluoroborophosphates reported so far (Table S1). And the calculation result of the bonding electron density differences (Δ*ρ*), based on the response electron distribution anisotropy (REDA) [50], shows that [BO_3_] units contribute 96% to the birefringence, confirming that the introduction of *π*-conjugated coplanar [BO_3_] units contributes greatly to the enhancement of birefringence (see Supplementary Fig. 9). As a result, the excellent comprehensive performance of ABPF, i.e. wide DUV transmittance, large SHG response and sufficient birefringence, is mainly derived from the unique KBBF-like structure composed of *π*-conjugated [BO_3_] units and non-*π*-conjugated [BO_4_], [PO_4_], [BO_3_F] units, which suggests the effectiveness of the multiple-anionic-groups design strategy. Moreover, the shortest PM wavelength of ABPF is 190 nm according to the calculation results of the refractive index dispersion curves (Fig. 3b), which suggests that ABPF has potential applications in the DUV field.

## CONCLUSION

In conclusion, a new type of KBBF-like compound, ABPF, with four different units, has been successfully obtained, and the synergistic effect of *π*-conjugated units and non-*π*-conjugated units means it exhibits excellent optical properties, namely, the highest NLO coefficients, the largest birefringence and the shortest PM SHG limit among all fluoroborophosphates. Owing to a beryllium-free, no-layered growth habit, and excellent optical properties, ABPF has a promising future as DUV NLO crystal. Moreover, we propose that the introduction of strong covalent bonds between layers can enhance the interlayer interaction force while simultaneously maintaining the large optical anisotropy of layered structures. More importantly, the emergence of ABPF once again proves the advancement of the ‘fluorination strategy’ in the DUV NLO field. These findings will facilitate the discovery of more DUV NLO materials with optimal and practical performance.

## METHODS

### Synthesis

Crystals were obtained via the high-temperature solution method in a closed system. NH_4_PF_6_ (95%, Aladdin), NH_4_HCO_3_ (AR, Aladdin) and B_2_O_3_ (98%, Kelong). All chemicals above were used without further purification. A mixture of NH_4_PF_6_ (0.502 g, 3.077 mmol), NH_4_HCO_3_ (0.608 g, 7.692 mmol) and B_2_O_3_ (1.285 g, 18.462 mmol) was loaded into a quartz tube (the inner diameter is 35 mm, and the length is 175 mm), and the tube was flame sealed under 10^−^^3^ Pa. The tube was heated to 400°C for 24 h, held at this temperature for 96 h, cooled to 300°C for 150 h and cooled to room temperature with a rate of 2°C h^−1^. Colorless crystals can be observed at the bottom of the tube, covered with a thin layer of amorphous sticky substance. After mechanical stripping, ABPF crystals can be obtained with the yield of ∼80% based on the B element.

### Characterizations

Powder XRD data were collected using a Bruker D2 PHASER diffractometer at room temperature. The single-crystal XRD data were collected using a Bruker D8 Venture diffractometer and the crystal structure was solved using Olex2. The interference pattern of polarized light was measured using a polarizing microscope (ZEISS Axioscope 5). TG-DSC were measured on a simultaneous NETZSCH STA 449 F3 thermal analyzer instrument under a flowing N_2_ atmosphere. The sample was placed in a Pt crucible and heated from 40 to 800°C at a rate of 5°C min^−1^. Elemental analysis was analyzed on the single crystal surface by a field emission scanning electron microscope (SEM, SUPRA 55VP) equipped with an energy dispersive X-ray spectroscope (EDX, BRUKER x-flash-sdd-5010). IR spectroscopy was measured by Shimadzu IR Affinity-1 Fourier transform infrared spectrometer. The transmittance measurement of a transparent crystal was measured by Shimadzu SolidSpec-3700DUV spectrophotometer under a flowing N_2_ atmosphere. Powder SHG intensity was measured via the Kurtz-Perry method using a Q-switched Nd: YVO_4_ solid-state laser (Cnilaser, DPS-1064-Q) at 1064 nm and 532 nm, for visible and UV SHG, respectively. Polycrystalline samples were ground and sieved into the following particle size ranges: 38–55, 55–88, 88–105, 105–150, 150–200 and 200–250 μm. The samples were loaded into a 1-mm-thick aluminum holder with an 8-mm-diameter hole. The sieved KDP and *β*-BBO samples were used as references.

## Supplementary Material

nwac110_Supplemental_Files
